# Impact of frailty status on clinical and functional outcomes after concomitant valve replacement and bipolar radiofrequency ablation in patients aged 65 years and older

**DOI:** 10.1186/s13019-022-02043-x

**Published:** 2022-11-28

**Authors:** Zhi-qin Lin, Xiu-jun Chen, Xiao-fu Dai, Liang-wan Chen, Feng Lin

**Affiliations:** 1 Department of Cardiovascular Surgery, Union Hospital, Fujian Medical University, Xinquan Road 29#, Fuzhou, 350001 Fujian People’s Republic of China; 2 Key Laboratory of Cardio-Thoracic Surgery (Fujian Medical University), Fujian Province University, Fuzhou, 350001 People’s Republic of China

**Keywords:** Older patients, Cardiac surgery, Quality of life, Age-related condition

## Abstract

**Background:**

To evaluate the prognostic value of frailty in older recipients of concomitant valve replacement (VR) and bipolar radiofrequency ablation (BRFA), we examined whether clinical and functional outcomes differed between frail and non-frail groups of older patients referred for concomitant VR and BRFA.

**Methods:**

In a single-center retrospective observational cohort study, we compared the clinical and functional outcomes in frail versus non-frail patients. Frailty was assessed using the 5-item Cardiovascular Health Study (CHS) frailty scale. Patients were divided into two groups, frail and non-frail. Functional outcome was assessed using the internationally validated Atrial Fibrillation Effect on QualiTy-of-Life (AFEQT) questionnaire.

**Results:**

We enrolled 185 patients aged ≥ 65 years who underwent concomitant VR and BRFA. About 36.2% (n = 67) of the patients were included in the frail group and the remaining patients (n = 118) in the non-frail group. Follow-up was complete with a median duration of 58 months (interquartile range 44–76 months). Significant differences were observed between the two groups with respect to postoperative pulmonary complications (frail vs. non-frail patients, 50.74% vs. 22.9%, respectively, *P* < .001) and hospital mortality (10.45% vs. 1.69%, respectively, *P* = .021). The frail group had a higher adjusted risk for all-cause mortality (adjusted HR 4.06; 95% CI 1.33 to 12.38; *P* = .014) and all-cause hospitalization (adjusted HR 2.24; 95% CI 1.12 to 4.50; *P* = .023). Frailty was associated with lower overall AFEQT scores at baseline (Estimate, − 0.400; 95% CI − 0.532 to − 0.267; *P* < .001). Compared to the non-frail group, the frail group continued to have lower overall AFEQT scores with no significant improvement in follow-up at 1 year and 2 years after concomitant VR and BRFA.

**Conclusion:**

Frail patients had lower baseline AFEQT scores and were more likely to have adverse outcomes from postoperative pulmonary complications, and frailty was also an independent risk factor for long-term all-cause mortality and all-cause rehospitalization. Further studies are needed to assess the impact of frailty.

## Background

Frailty is common in adults aged 65 years and older, with a prevalence ranging from 4 to 16.2% [1]. Frailty syndrome describes an age-related condition of increased vulnerability that is characterized by reduced physiological reserve and greater vulnerability to adverse health outcomes [2]. Atrial fibrillation (AF) and frailty often occur together, and their incidence increases progressively with age, making them the central concerns in an aging society [3, 4]. Whether concomitant bipolar radiofrequency ablation (BRFA) should be performed in older patients remains controversial. Many studies support the use of concomitant valve replacement (VR) and BRFA in the treatment of older patients with valve disease combined with AF [7, 8]. However, some studies suggest there is little or no benefit from concomitant BRFA for a proportion of these patients [9, 10]. Many surgeons are still reluctant to perform concomitant AF ablation in older patients because of concerns that this procedure may increase overall surgical risk.

However, age alone does not fully explain the increased risk of concomitant ablation procedures. Frailty may be one of the contributing factors in high-risk older patients. A number of studies have explored the relationship between frailty and increased risk of cardiovascular diseases among older people [5, 6]. There is currently no consensus on the exact criteria for determining whether to perform BRFA concomitant with VR. To our knowledge, few analyses have focused on whether frailty status should be a factor in determining whether to perform concomitant BRFA, and the impact of frailty in this population is unknown. Our center prospectively collects information on surgical treatment of valve disease combined with AF and patients’ health status. Therefore, in this study, we sought to evaluate the prognostic value of frailty in older recipients of concomitant VR and BRFA, and we examined whether clinical and functional outcomes differed between frail and non-frail groups of older patients referred for concomitant VR and BRFA.

## Material and methods

### Study population

We conducted a retrospective observational cohort study of prospectively collected data from a total of 235 consecutive older patients (aged ≥ 65 years) who underwent concomitant VR and BRFA between January 2011 and March 2020 at our institution. Ethics approval with a waiver of informed consent was obtained from the ethics committee of Union Hospital, Fujian Medical University (ID number: 2021KY182). Detailed clinical information and follow-up results had been entered prospectively into a dedicated database for subsequent analysis. Inclusion criteria: (1) Patients with biological valves; (2) patients who had persistent or long-lasting persistent AF before surgery. Exclusion criteria: (1) Patients with mechanical valves; (2) patients who underwent other surgery involving the aorta, coronary arteries, and so on. The primary outcome measure was all-cause mortality during follow-up. Based on our previous observations, the sample size was calculated using an online sample calculator (https://epitools.ausvet.com.au/samplesize). Based on a two-sided type 1 error of 0.05 and a power of 0.80, a required minimum sample size of 41 patients in the frail group and 82 in the non-frail group was calculated. To minimize any effect of data loss, 185 patients were included in the final sample. The diagnosis of AF was confirmed by 24-h Holter monitoring before surgery. Frailty assessment was conducted on the day of admission as a routine preoperative screening.

## Measurements and outcomes

### Frailty scales

The preoperative frailty status was determined by a trained medical professional according to the 5-item Cardiovascular Health Study (CHS) frailty scale (gait speed, handgrip strength, physical activity, exhaustion, weight loss). The details of the CHS frailty measurements have been reported previously [11]. Frailty was considered present if the subjects had met three of these five components. Gait speed was assessed by the 5-m walk test. A slow 5-m walk test was defined as completing the test in > 6 s or being unable to complete the test. Patients who normally require a walking assistive device were allowed to use them during the test. Handgrip strength was assessed with a hand-held dynamometer (EH101. CAMRY, Guangdong, China). Weak grip strength was defined as the average value from three measurements < 18 kg in women and < 30 kg in men [12]. Physical activity was calculated according to an 18-item activity survey based on the short version of the Minnesota Leisure Time Activity questionnaire. Exhaustion was measured using self-answers to two questions taken from the Center for Epidemiologic Studies Depression Scale (CES-D). Weight loss was defined as unintentional weight loss of more than 0.454 kg (10 pounds) within one year prior to surgery.

### Atrial fibrillation effect on QualiTy-of-life (AFEQT) score

Functional outcomes were assessed by determining the quality of life (QoL) at baseline, the 1-year follow-up visit, and the 2-year follow-up visit, using the internationally validated AFEQT measure (https://www.afeqt.org). The AFEQT, used widely in AF research, is a 20-item questionnaire measuring four domains of patient health status, namely symptoms (n = 4), daily activities (n = 8), treatment concern (n = 6), and treatment satisfaction (n = 2). The AFEQT score can range between 0 and 100, with a lower score reflecting a worse functional outcome.

### Clinical outcome measures

Clinical outcomes were obtained from patients’ medical records at our outpatient clinic or by telephoning patients or their caregivers. Analyses of in-hospital mortality and postoperative complications (postoperative pulmonary complications included postoperative pneumonia and postoperative respiratory failure, other postoperative complications defined according to the Society of Thoracic Surgeons as stroke, renal failure, prolonged ventilation, or need for reoperation) were performed [13]. Additionally, length of intensive care unit (ICU) stay, ICU readmission, length of hospital stay, and hospital stay were reported. Late clinical outcomes assessed in follow-ups included all-cause mortality, cardiovascular death, stroke or non-central nervous system (non-CNS) embolism, AF recurrence, all-cause hospitalization, and cardiovascular hospitalization. Cardiovascular death was defined as death due to malignant arrhythmia, stroke, or congestive heart failure. Cardiovascular hospitalization was defined as hospitalization due to cardiovascular disease. Survival was measured as time (months) to death from the date of surgery. AF recurrence was defined as any episode of AF, atrial flutter, or atrial tachycardia lasting more than 30 s recorded by electrocardiogram or 24-h Holter monitoring after six months of surgical ablation. The period between the next day after patients received surgical operation and death, loss to follow-up, or the predefined date of June 2020 was defined as the follow-up duration.

### Surgical procedures and postsurgical treatment

VR with concomitant BRFA was initially performed under general anesthesia with hypothermic cardiopulmonary bypass through a median sternotomy. The surgical technique details and postoperative treatment (anti-arrhythmic therapy, control of ventricular rate, and anticoagulation) have been described in our previous paper [14]. The Medtronic Cardioblate G2 Surgical Ablation System (Medtronic Inc., Minneapolis, MN) with a bipolar radiofrequency clamp was used for surgical ablation. After surgical procedures, patients were admitted to the ICU for early monitoring before being transferred to the general cardiology ward.

### Statistical analysis

Continuous variables are expressed as mean ± standard deviations and were compared using the Student’s t-test or Mann–Whitney U test. Continuous variables are summarized as medians (interquartile ranges [IQRs]) and were compared using the Wilcoxon rank-sum test. Categorical variables are presented as counts or proportions and were compared by the chi-square test. Overall survival and AF-free survival were summarized using the Kaplan–Meier method. To examine the association between frailty status and overall AFEQT score at baseline or change in overall AFEQT score, a multivariable hierarchical linear regression model was constructed using a stepwise selection with a stay criterion of α = 0.05 for selecting covariates. Change in overall AFEQT score was defined as the overall AFEQT score at the time of the review minus the overall AFEQT score at baseline.

Subsequently, to determine whether frailty status was independently associated with outcomes, Cox regression models were used to examine the association of frailty status and time from enrollment with outcomes in follow-up (all-cause death, cardiovascular death, revascularization, stroke or non-CNS embolism, all-cause hospitalization, cardiovascular hospitalization). Multivariate Cox regression models were adjusted according to all covariates listed in the appendix of Table 3 with a retention criterion of α = 0.25. Hazard ratios (HR) and 95% confidence intervals (CI) were computed. Fine and Gray competing risk regression was used to evaluate the impact of frailty status on the risk of cardiovascular death, with non-cardiac death as the competing event. Multivariate competing risk regression models were adjusted according to all covariates listed in the appendix of Table 3 with a retention criterion of α = 0.15. The subdistribution hazard ratio (SHR) and 95% CI were computed. A two-sided *P* value < 0.05 was considered statistically significant. The statistical software used throughout the analysis was SPSS 26.0 and R 4.0.3.

## Results

The baseline characteristics of the cohort stratified by baseline frailty status are shown in Table 1. Follow-up was complete with a median duration of 58 months (IQR 44–76 months). About 36.2% of the study patients were identified as frail. Between the frail and non-frail groups, significant differences were observed in the parameters age, sex, body mass index, hypoalbuminemia, left ventricular ejection fraction, and New York Heart Association (NYHA) functional class (all *P* < 0.05).

**Table 1:** Comparison of patients’ baseline demographic and clinical characteristics

Variables	Total sample (n = 185)	Patient groups		
		Frail (n = 67)	Non-frail (n = 118)	*P* value
Age (yr)	70.52 ± 4.51	69.58 ± 3.87	71.06 ± 4.76	0.023
Male (n)	98	28	70	0.022
BMI (kg/m^2^)	21.92 ± 2.92	21.62 ± 3.12	22.21 ± 2.47	0.182
Normal BMI: 18.5–23.9 kg/m^2^ (n)	106	30	76	0.009
Hypoalbuminemia (n)	19	11	8	0.038
Smoking history (n)	48	23	25	0.050
Diabetes (n)	23	7	16	0.538
Hypertension (n)	88	33	55	0.729
Peripheral vascular disease	14	5	9	0.968
LVEF	0.615 ± 0.079	0.589 ± 0.086	0.630 ± 0.071	0.001
NYHA (IV) (n)	20	15	5	0.000
AF duration (years)	7.39 ± 3.96	7.06 ± 3.53	7.58 ± 4.18	0.387
eGFR, mL/min/1.73 m^2^	96.61 ± 17.52	94.74 ± 16.78	97.67 ± 17.91	0.276
COPD(n)	7	1	0	0.314
Cancer(n)	6	2	4	0.881
Severe pulmonary hypertension (n)	36	15	11	0.448
Stroke or non-CNS embolism (n)	28	10	18	0.952
CAD(n)	26	11	15	0.486
liver dysfunction (n)	18	8	10	0.445
*The number of valves requiring surgery (n)*				
Double	41	12	29	0.627
Single	144	55	89	

BMI, body mass index; LVEF, left ventricular ejection fraction; NYHA, New York Heart Association; COPD, chronic obstructive pulmonary disease; TIA, Transient Ischemic Attack; CAD, coronary artery disease

### Operative data and early outcomes

Table 2 shows the operative data and early outcomes. The frail group had a significantly longer duration of ICU stay (9.25 ± 5.36 days vs. 6.37 ± 4.24 days, *P* < 0.001) and a slightly increased length of hospital stay (18.09 ± 7.16 days vs. 15.53 ± 5.30 days, *P* = 0.012) than the non-frail group.

**Table 2:** In-hospital outcomes

Variables	Total sample (n = 185)	Frail (n = 67)	Non-frail (n = 118)	*P* value
Prolonged mechanical ventilation (> 24 h) (n)	22	13	9	0.017
Tracheostomy	6	5	1	0.044
Pulmonary complication (n)	61	34	27	0.000
Stroke or non-CNS embolism (n)	16	8	8	0.230
Renal failure (n)	5	2	3	1.000
Ventricular arrhythmias	17	9	8	0.132
ICU stay (days)	7.42 ± 4.86	9.25 ± 5.36	6.37 ± 4.24	0.000
ICU readmission (n)	13	8	5	0.049
Length of hospital stay (days)	16.46 ± 6.14	18.09 ± 7.16	15.53 ± 5.30	0.012
Low cardiac-output (n)	6	4	2	0.252
Reoperation for bleeding (n)	3	2	1	0.617
Permanent pacemaker (n)	3	1	2	1.000
Hospitalized death cases (n)	9	7	2	0.021

ICU, intensive care unit; CNS, non-central nervous system

Significant differences were also observed among the groups with respect to pulmonary complications (50.74% vs. 22.9%, *P* < 0.001). More patients in the frail group required prolonged mechanical ventilation. In the frail group, 13 of 67 patients required prolonged mechanical ventilation (> 24 h) compared with 9 of 118 patients in the non-frail group (19.40% vs. 7.63%, respectively, *P* = 0.017), and 5 of 67 patients required tracheostomies compared with 1 of 118 patients (7.46% vs. 0.85%, respectively, *P* = 0.044). Hospital mortality was higher in the frail group than in the non-frail group (10.45% vs. 1.69%, *P* = 0.021). Seven patients died in the frail group (hematencephalon in 1 case, malignant arrhythmia in 1 case, severe pneumonia in 4 cases, low cardiac output syndrome and multiple organ failure in 2 cases), and 2 patients died of pneumonia in the non-frail group (hematencephalon in 1 case, severe pneumonia in 1 case).

Other adverse events such as neurological complications, ventricular arrhythmias, renal failure, low cardiac output, reoperation for bleeding, and pacemaker implantation were similar between both groups.

### Late clinical outcomes

**Fig. 1:**
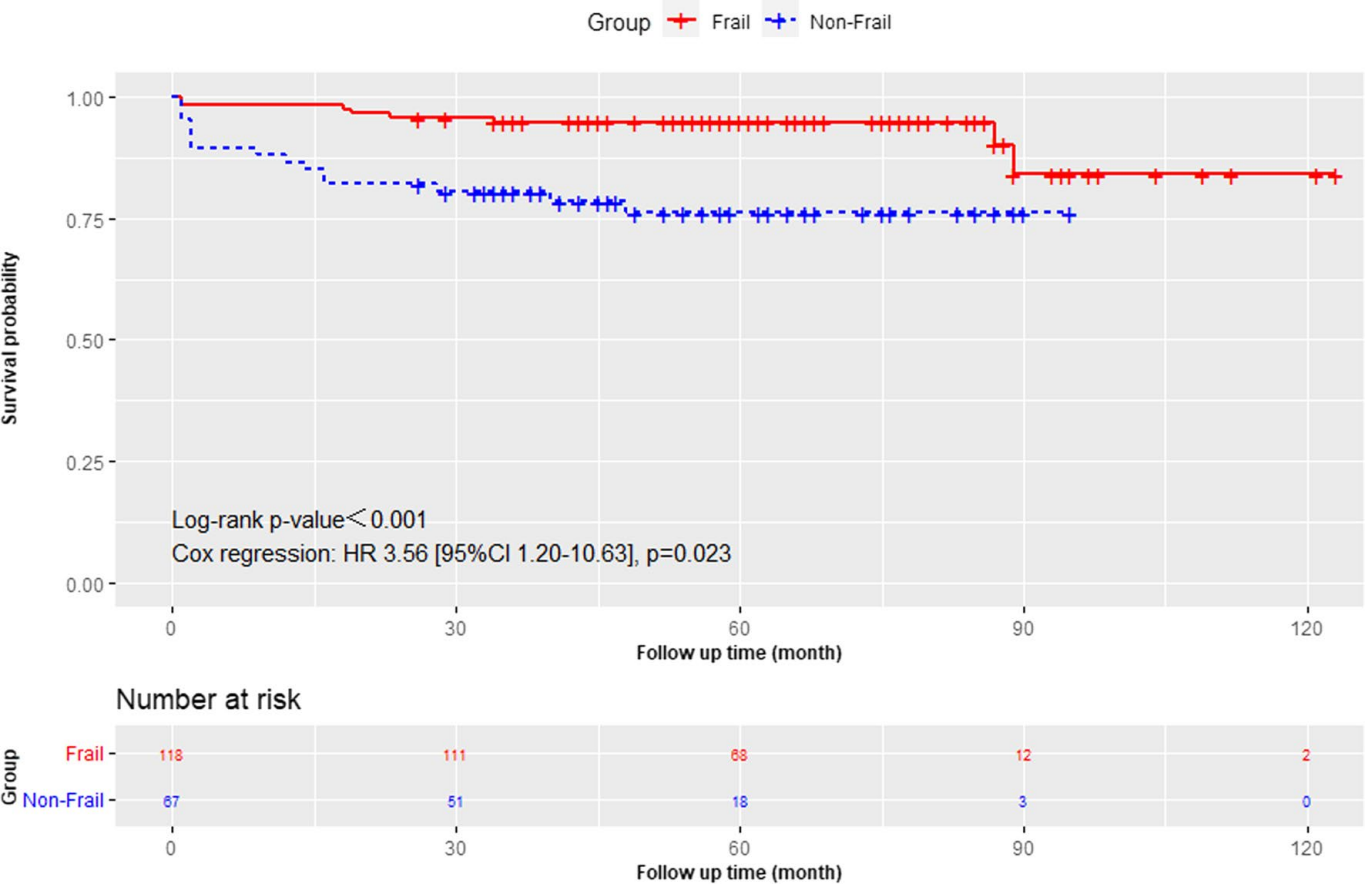
The survival of the frail and non-frail groups

**Fig. 2:**
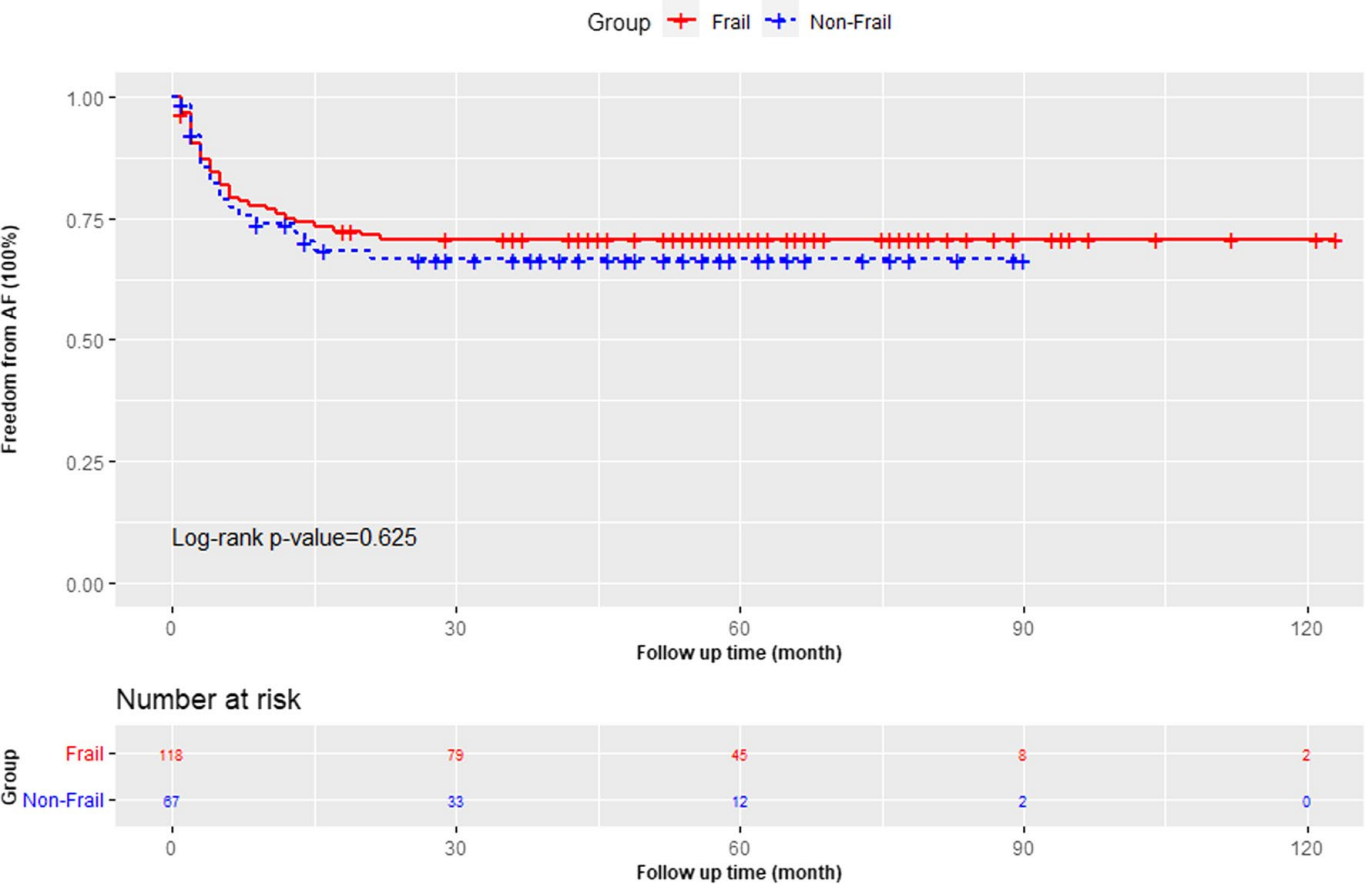
Freedom from AF during follow up of frail and non-frail groups

Kaplan–Meier analysis revealed a significantly worse overall survival (*P* = 0.002) in the frail group during clinical follow-up (as shown in Fig. 1). The overall sinus rhythm rate at the latest follow-up was 69.4%. The Kaplan–Meier survival curves for freedom from AF showed no significant difference between the two groups (as shown in Fig. 2). Freedom from AF after 1 year was 75.1% in the frail group compared to 73.8% in the non-frail group. Full follow-up outcomes in the frail group compared with those in the non-frail group are shown in Table 3. After accounting for baseline risk, the frail group had a higher adjusted risk for all-cause mortality (adjusted HR 4.06; 95% CI 1.33 to 12.38; *P* = 0.014) and all-cause hospitalization (adjusted HR 2.24; 95% CI 1.12 to 4.50; *P* = 0.023). The observed rates of cardiovascular death, stroke or non-CNS embolism, and cardiovascular hospitalization were similar between the two groups.

**Table 3:** Univariable and multivariable analyses of outcomes in full follow-up according to the frailty status

Outcomes	No. of events/total number of patients		Univariable		Multivariable	
	Frail	Non-frail	HR (95% CI)	*P* value	HR (95% CI)	*P* value
All-cause death^a^	15/67	7/118	4.00 (1.69–9.49)	0.002	4.06 (1.33–12.38)	0.014
Cardiovascular death^b^	7/67	3/118	3.26 (0.78–13.60)	0.110	2.85 (0.62–13.10)	0.180
Stroke or non-CNS embolism^c^	7/60	7/116	4.25 (1.38–13.07)	0.011	2.21 (0.68–7.17)	0.186
All-cause hospitalization^d^	16/60	18/116	2.47 (1.25–4.87)	0.009	2.24 (1.12–4.50)	0.023
Cardiovascular hospitalization^e^	11/60	8/116	3.46 (1.38–8.67)	0.008	2.56 (0.92–7.11)	0.070

CNS, non-central nervous system

^a,b,c,d,e^Adjusted for baseline covariates: age, gender, normal BMI, LVEF, NYHA (IV), hypoalbuminemia, smoking history, diabetes, hypertension, peripheral vascular disease, hypohepatia, COPD, Cancer, severe pulmonary hypertension, stroke/TIA, CAD, liver dysfunction, the number of valves requiring surgery, frailty

### Functional outcomes

**Fig. 3:**
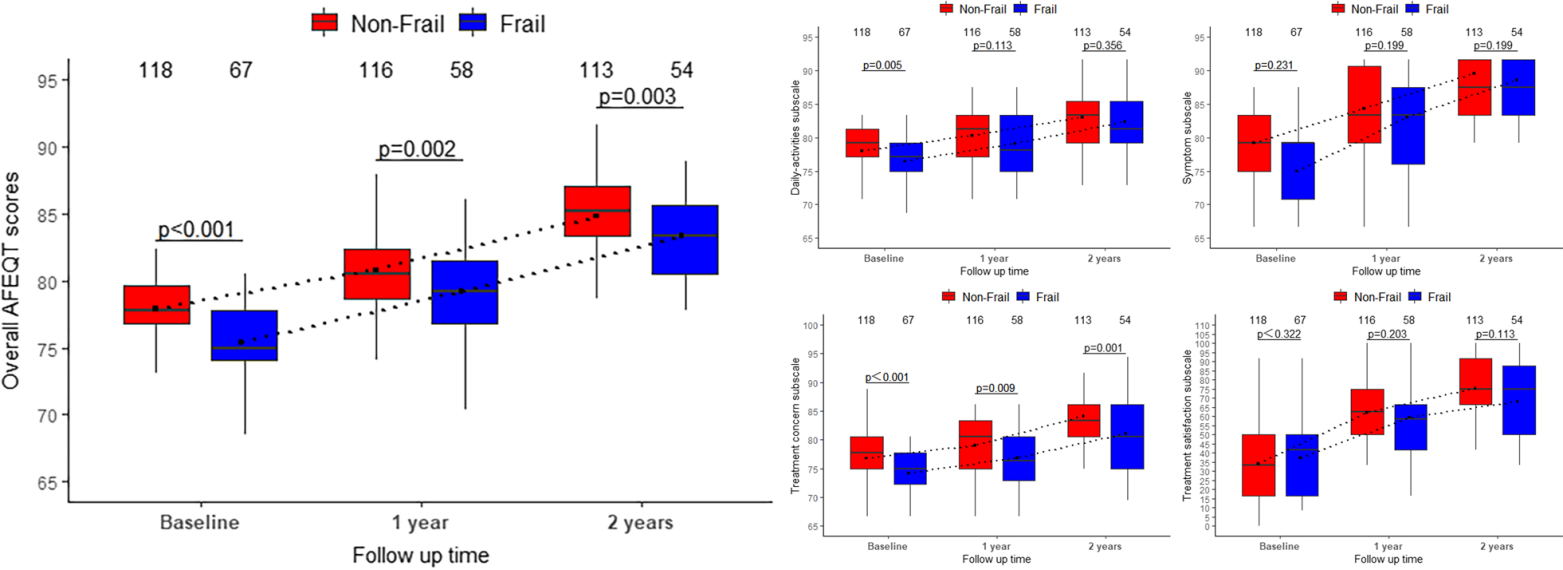
Comparison of the AFEQT score between the frail and non-frail groups

In the health-related QoL substudy, the frail group had lower overall AFEQT scores at baseline compared to the non-frail group (75.00; IQR 74.07–77.78 vs. 77.78; IQR 76.85–79.63; *P* < 0.001; Table 4). As shown in Fig. 3, the frail group had consistently lower AFEQT domain scores including symptoms, daily activities, and treatment concerns. After adjustment for important characteristics, frailty was associated with lower overall AFEQT scores (Estimate, − 0.400; 95% CI − 0.532 to − 0.267; *P* < 0.001). Treatment satisfaction at baseline was similar in the frail and non-frail groups (41.67; IQR 16.67–50.00 vs. 33.33; IQR 16.67–50.00; *P* = 0.323). One-year follow-up QoL data were available for 174 (94.1%) patients, and 2-year follow-up QoL data were available for 168 (90.8%) patients. When follow-up QoL at 1 year was investigated among 174 patients, the frail group continued to have lower overall AFEQT scores with no significant improvement in follow-up vs. the non-frail group (Estimate, 0.071; 95% CI − 0.104 to 0.245; *P* = 0.424), and this persisted out to 2 years (Estimate, 0.103; 95% CI − 0.073 to 0.279; *P* = 0.248).

**Table 4:** AFEQT scores at baseline and changes in follow-up for frail vs non-frail

Outcome	No	Unadjusted		Adjusted^a^	
		Estimate (95% CI)	*P* value	Estimate (95% CI)	*P* value
Overall AFEQT score at baseline^b^	185	− 0.444 (− 0.574 to − 0.313)	< 0.001	− 0.400 (− 0.532 to − 0.267)	< 0.001
Change in AFEQT score at 1 y^c^	174	0.127 (− 0.023 to 0.276)	0.096	0.071 (− 0.104 to 0.245)	0.424
Change in AFEQT score at 2 y^c^	168	0.143 (− 0.008 to 0.295)	0.065	0.103 (− 0.073 to 0.279)	0.248

AFEQT, Atrial Fibrillation Effect on QualiTy-of-life

^a^Adjusted for baseline covariates in overall AFEQT baseline model and change in AFEQT score models (at 1 year and 2 years): age, gender, normal BMI, LVEF, NYHA (IV), hypoalbuminemia, smoking history, diabetes, hypertension, peripheral vascular disease, hypohepatia, COPD, Cancer, severe pulmonary hypertension, stroke/TIA, CAD, liver dysfunction, the number of valves requiring surgery, frailty

^b^Estimate represents difference in overall AFEQT score at baseline

^c^Estimate represents difference in mean change

## Discussion

Frailty has been found to be a potential predictor of increased adverse outcomes in older cardiac surgical populations [15]. In this study, we analyzed the effects of frailty on older patients who underwent concomitant VR and BRFA. There were four main findings. First, a significant proportion of the study patients had a combination of frailty. Second, frail patients tended to have poorer cardiac functions. Third, during hospitalization, frailty affected the duration of ICU stay and hospital stay for VR with concomitant BRFA procedures. Moreover, frail patients had a higher risk of pulmonary complications. Finally, in terms of late clinical outcomes, frail patients had higher risk-adjusted all-cause mortality and all-cause hospitalization rates.

Frailty is considered a general indicator of patient vulnerability and is highly associated with adverse health outcomes in the field of geriatrics [16], and it is gaining traction in the field of cardiac surgery [17]. With the development and promotion of AF surgical radiofrequency ablation technology in recent years, valve surgery concurrently with AF surgical radiofrequency ablation technology has obvious advantages, and successful conversion to sinus rhythm eliminates the burden of symptoms of AF after surgery for valvular heart disease. The overall sinus rhythm rate at the latest follow-up was 69.4%, which was comparable to that calculated in previous studies. Nakamura et al. reported that the rate of freedom from AF in 143 patients (age 65 years or older) with concomitant surgical ablation was 74.9% [18]. The question of whether to perform BRFA in older patients with valvular disease combined with AF simultaneously has been previously explored in several studies [7–10], including one of our own [14], and some well-known risk factors, such as age, body mass index, and left atrial size, have been shown to adversely affect patients undergoing heart valve surgery combined with AF ablation [19–21]. However, to our knowledge, comparative clinical data on risk stratification using a frailty index in older patients undergoing surgical radiofrequency ablation techniques for concurrent AF are lacking. Surgeons often refuse to perform concomitant BRFA because of the patient's frailty.

Previous studies have shown that age, AF, cardiovascular disease, and other factors are associated with the degree of frailty [3, 22–24]. Yang et al. used the CHS frailty phenotype and the Edmonton Frailty Scale to detect frailty in approximately 8.3% of older patients (> 65 years of age) with AF [4], whereas our retrospective observational study showed that frailty was higher than in their study, with 36.2% of older patients hospitalized with AF. This may be because our study patients all had valvular diseases resulting in an increased proportion of frail patients. Frail patients reported worse cardiac function, suggesting that there may be an association between the degree of cardiac function and frailty status. One possible explanation is that cardiac insufficiency may lead to decreased mobility, fatigue, poor appetite, and weight loss, which subsequently leads to weakness in frail patients. In our multivariate logistic regression and Cox proportional survival analyses, we included many potential confounding variables, such as cardiac function class, as covariates. This essentially eliminated the effect of these potential confounders and further validated the effect of frailty status on surgical outcomes in older patients with valve disease combined with AF. We found that frail patients had prolonged hospital stay and postoperative time in the ICU. This is similar to the reports of previous studies that used various frailty criteria [25, 26]. This may be partly attributed to the fact that in our study, postoperative pulmonary complications occurred significantly more in frail patients than in non-frail patients, which significantly prolonged the length of hospital stay and time in the ICU [27].

In older patients, we need to balance the risk of intervention with the improvement of QoL [28]. Previous studies have shown that frailty is an independent predictor of increased intensity of arrhythmia symptoms and worsening QoL [29]. In our study, we used a disease-specific method of QoL assessment, the AFEQT questionnaire, to assess patients with AF. After adjusting for important patient characteristics, adjusted baseline QoL was also found to be significantly lower in frail patients than in non-frail patients. This may be due to the fact that the current assessment of QoL in patients with AF is highly dependent on the patient's symptom status, and frailty may make patients more sensitive to AF symptoms and perceive disease symptoms differently. In addition, analysis of the component domains of AFEQT showed that at baseline, frail patients had more severe symptoms, were more limited in their daily activities, and had more concerns about their treatment. Therefore, when analyzing the impact of radiofrequency ablation on QoL in older patients, clinicians should be aware of these differences and ensure that symptom burden and QoL at baseline are carefully assessed before selecting a treatment strategy. When follow-up QoL was investigated, patients of the frail group continued to have lower overall AFEQT scores with no significant improvement in follow-up compared to those of the non-frail group. Direct comparison of preoperative and postoperative QoL in older patients is limited by the presence of censored data, which requires further study.

Predicting early and late clinical outcomes and risks can help inform treatment decisions. However, current cardiac surgery risk models, including the EuroSCORE II and STS risk models, play an important role in predicting early cardiac surgery outcomes including hospital mortality, but they often fail to directly predict long-term outcomes [30]. These risk models often do not take into account a comprehensive assessment of frailty, which may be an important determinant of the outcome, particularly in older patients. An important implication of correctly identifying frailty is how to provide the appropriate surgical treatment modality for older patients with valvular disease combined with AF. Although our previous study suggested that this technique could provide clinical benefit to older patients, in practice, most surgeons do not consider older patients for simultaneous BRFA procedures.

In our study, frailty was assessed based on the patient's status prior to hospitalization, and we found an increased risk of early postoperative pulmonary complications and in-hospital death in the frail population. In the late clinical outcome of our study, survival was significantly lower in patients in the frail group who were discharged after concomitant BRFA with VR than in those in the non-frail group. Frailty was also identified as a risk factor for all-cause mortality according to univariate and multivariate analyses. Of note, we found it interesting that although the adjusted risk of all-cause mortality and all-cause hospitalization risk for frail patients were 3.06 and 2.36 times higher than those for non-frail patients, respectively, adjusted stroke incidence, cardiovascular-related mortality, and rehospitalization for cardiovascular causes were not significantly different between frail patients and non-frail patients. Therefore, the characteristics of readmission burden, stroke incidence, and cardiovascular-related mortality in frail patients mentioned in this study need to be considered in future evaluations of frail patients undergoing concomitant BRFA with VR in older patients. Our findings suggest that frailty assessment tools may provide valuable information for preoperative communication with patients about their condition, preoperative stratification of risk for late mortality, prediction of major in-hospital adverse events, QoL, and late clinical outcomes.

However, it is also important to highlight that many frail patients underwent surgery without experiencing poor outcomes. Future research should focus on reducing the impact of frailty on clinical outcomes, and teams involved in performing VR and BRFA procedures need to be skilled in managing frail patients. Surgeons should be aware that a significant proportion of older patients who undergo VR and BRFA may be frail and establish with geriatricians the practice of preoperative comprehensive geriatric assessment, postoperative management of frail patients, and improvement in postoperative QoL. With a closer working relationship, specific care models should be developed in the future for this population to reduce the incidence of frailty and improve the clinical outcomes in this patient population. Our finding also suggests that an accurate preoperative frailty assessment may provide a better risk–benefit assessment for each patient, which may have important implications for Medicare and Medicaid costs. Furthermore, there is a need to focus on how treatments and interventions specifically affect all-cause outcomes in frail patients and predict the discharge of frail patients in order to improve discharge planning and coordinate health care costs and resource use. These should be confirmed in further studies.

Our study has limitations that are inherent to retrospective studies. We did not include vulnerable patients who were considered to be "inoperable" by their surgeons and whose frailty may have been quite severe. We cannot extrapolate what the clinical outcome of these patients would have been if they had undergone simultaneous BRFA procedures. Therefore, clinicians should consider factors such as further grading of frailty before interventions in older patients with AF considered too debilitated to review the indications for surgery. Additionally, in clinical practice, there is currently a lack of consensus regarding the best tool to assess frailty. Although the CHS frailty assessment method is easy to administer and perform and is a fairly simple qualitative assessment tool, its inability to indicate the degree of frailty through scoring makes it possible that our findings do not fully reflect the impact of frailty. More patients and a longer follow-up period with testing of other possible definitions of frailty are needed to further confirm the observed effects. In addition, the benefit in patients who did not recover sinus rhythm may not be as significant as in patients who recovered sinus rhythm. In our hospital, older patients over the age of 65 years have relatively rarely chosen mechanical valves. Since thromboembolic events are common after mechanical valve replacement, we excluded patients with mechanical valve replacement to more accurately compare the occurrences of postoperative strokes in the two groups. However, further large-scale studies are needed to address this issue. Nevertheless, our current findings may provide the premise for hypothesis generation and may serve as hypothetical information for future prospective studies to clarify the impact of preoperative frailty status on VR combined with BRFA.

## Conclusion

Our study shows that frailty assessed according to the CHS method is common in older patients with valve disease combined with AF. Frail patients have lower baseline AFEQT scores and are more likely to have adverse outcomes from postoperative pulmonary complications. Frailty is also an independent risk factor for long-term all-cause mortality and all-cause rehospitalization. However, the risks of long-term cardiovascular-related mortality, rehospitalization for cardiovascular causes, stroke or systemic embolism, and recurrence of AF in frail patients are similar to those in non-frail patients. Further studies are needed to assess the impact of frailty.

## Data Availability

All data generated or analyzed during this study are included in the published article.

## Abbreviations

AF: Atrial fibrillation
BRFA: Bipolar radiofrequency ablation
HR: Hazard ratio
SHR: Subdistribution hazard ratio
CI: Confidence interval
VR: Valve replacement
ICU: Intensive care unit
IQR: interquartile range
CNS: Central nervous system
AFEQT: Atrial Fibrillation Effect on QualiTy-of-life
QoL: Quality of life
CHS: Cardiovascular health study
